# Making lighting adjustments to establish new behavioral patterns in a child with autism: A follow-up study

**DOI:** 10.22037/ijcn.v16i2.26907

**Published:** 2022-03-14

**Authors:** Emily PIVEN, Seyed Alireza DERAKHSHANRAD

**Affiliations:** 1 University of St. Augustine for Health Sciences, St. Augustine, Florida, USA; 2 Department of Occupational Therapy, School of Rehabilitation Sciences, Shiraz University of Medical Sciences, Shiraz, Iran

**Keywords:** Attention, Lighting, Occupational Therapy, Training Activity

## Abstract

Acknowledging the importance of lighting adjustment (a less-studied aspect of the environmental modification), this study showed novel effects of black light conditions, where white objects became part of the foreground of a blackened environment to train a child with autism to master a series of self-care tasks. This follow-up study provided details about how training progressed under black light conditions to teach the child a second task called self-feeding. The process of training self-feeding for this child was undergone after the child mastered the self-care task of toothbrushing. Healthcare practitioners may want to illuminate overlooked aspects of the non-human environment, which may be ignored by children with autism, to stimulate interest in objects following lighting adjustments.

## Introduction

Autism spectrum disorder (ASD) is a neurodevelopmental disorder associated with patterns of behaviors manifested through a range of traits showing hyperactivity or hypoactivity in reaction to sensory input, as well as inappropriate inter- and intrapersonal responses to social communication (1). The current trend in the treatment of ASD is toward the necessity of considering the complex interaction between personal and environmental factors, known as underlying determinants of children’s behavioral manifestations (2, 3). This multifactorial perspective suggests that the characteristics or personal factors of a child with autism (i.e., motivation, meaningful experiences, communication, and interpersonal abilities), along with features of his/her environment (i.e., environmental supports), become determinants of behavioral patterns that can affect function. This paper explained how the backlighting of the physical environment might help establish new behavioral patterns in a child with autism. We described an innovative approach of using a black light to modify the environment to enhance the meaning-making process in the brain, thus helping the child get attention to interact with objects for the development of independence in self-feeding.

Although nothing was noted about lighting adjustments, a systematic review of occupational therapy interventions for children with feeding disorders, such as delay in self-feeding, found strong to moderate evidence in support of environmental modifications to enhance self-feeding performance (4). By reviewing the literature, the authors emphasized pursuing three important occupational therapy goals to solve feeding-related issues, which address improving (a) self-feeding skills, (b) oral-motor skills, and (c) food acceptance patterns (4). More specifically, in order to address issues related to delayed self-feeding, it was recommended that a developmental training protocol of self-feeding skills should be established using assistive feeding devices to teach the child how to, for example, hold a spoon, scoop food, or bring a spoon to mouth (4). It is now recognized that children with ASD would considerably benefit from environmental adjustments that modify tasks in the expectation of facilitating learning new behavioral modes (5). In doing so, the child presented in this report was taught how to self-feed using an individualized education plan (IEP), which encompassed a special task modification in combination with a special instructional approach. IEP included (a) incorporating black lighting to enable the child to focus on white food and white feeding devices that appeared against a black background and (b) application of the Try Another Way (TAW) methodology to provide the child of limited learning capacity a unique opportunity whereby she received an instructional program that had her achieve independence incrementally (6). Based on the belief that everyone can learn, TAW is a systematic training approach using task analysis to figure out how to teach functional skills to developmentally disabled persons difficult to train . Another Way Methodology. Retrieved 2021/12/01, from Marc Gold & Associates. http://www.marcgold.com/. 

## Case Presentation

The Institutional Review Board at the University of St. Augustine for Health Sciences, Florida, USA, approved reporting this case. Mary (fictitious name) resided at a private residential school for children with autism located in a small town in the United States, from 5 to 8 years of age. She appeared to be a normal toddler until 14 months, when she was diagnosed with autism by a pediatric psychiatrist. She had lost all interest in objects in her surrounding environment. It could not be determined whether regression was because of a lack of motivation, a visual discrimination problem, or other factors. Mary stood with a wide base of support and rocked most of the day with tightly clasped hands as she privately smiled to herself with downcast eyes. She was a passive participant in every basic daily activity. Staff would give total physical assistance for all daily routines. She showed no emotion or resistance to the passive participation in daily self-care activities of bathing, toileting, dressing, undressing, hair washing, drying with a blow dryer, hair brushing or combing, toothbrushing, and eating. If she fell and scraped her hands or knee, she was oblivious. One day during lunch, it was discovered that Mary liked popcorn when she reached into a bowl of popcorn on the table and put a fist full into her mouth. Thereafter, she continued to pick up popcorn that she found anywhere in the environment, and it became the basis of behavioral reinforcement. The psychologist placed Mary on an operant conditioning program to shape Mary’s eye contact using a piece of popcorn as positive reinforcement.

The first author, an occupational therapist, successfully taught Mary to brush her teeth under black light conditions (7). The school psychologist referred Mary once again to assess and decide whether she might benefit from additional training in other areas of self-care. The facility staff were excited to help determine if Mary would be able to develop self-feeding skills. As a result, this was a follow-up study to investigate the generalizability of the black lighting approach to teach the child a second self-care activity. Accordingly, the similarity of this brief report to the previously published paper is that (a) it was the same child, (b) the same training aids, and (c) the same instructional method fully described in the previously published paper (7).

During the previous 4-month period, Mary received training 3 times/day under black light conditions, which brought attention to the illuminated elements of toothbrushing activity, including her white teeth, white sink, and other related objects (8). After learning how to brush teeth under black light conditions, she initiated interaction with objects, ventured to experience new choices of actions, and started to assign meaning to other self-care activities, assimilating them as meaningful involvement. Subsequently, research questions were as follows: 

I. Could Mary be trained under different black light environments to become independent at a new task, thus enhancing her sensory experiences of taste, smell, touch, kinesthetic, proprioceptive, vision, and hearing senses while focusing on foods and related objects, which could be reinforced by the sensory experiences of self-feeding? 

II. Would she generalize drinking from a white cup under a new condition? 

III. Upon reaching criterion, could Mary sustain her interest in self-feeding with standby assistance when necessary?

For one week, she was observed, and frequency data were collected every day and evening to determine her baseline of self-feeding at meals. She had no incidences of self-feeding at baseline and no interest in exploring food on the plate. Mary was not interested in bringing food placed in her hand to her mouth (such as bread, cornbread, muffins, crackers, carrot sticks, celery with peanut butter, grapes, banana, apple slices, pineapple, coconut, raisins, chocolate chip cookies, oatmeal bars, etc.). Likewise, when utensils were placed in her hand with meat, vegetables, fruit, cake/pudding, or hot/cold cereal, she did not initiate action toward her mouth. Mary failed to interact with objects because she did not find meaning or stimulation in usual self-feeding tasks. However, she would open her mouth, lateralize, and chew food fed to her and drink from a cup. In addition, there was no food that she expressed for. 

On her IEP, it stated that during mealtime, Mary was supposed to sit at an adapted table with a scoop out for a chair, a black heavy plastic plate with a white plastic plate guard, and a cylindrical white cup with a trained aide for standby assistance. The goals were as follows: (a) Using faded physical, verbal, gestural, and demonstration prompts, Mary will initiate hand to mouth self-feeding with standby assistance and (b) using faded physical, verbal, gestural, and demonstration prompts, Mary will initiate spoon to mouth self-feeding behavior until she is 100% independent in self-feeding three meals per day for 90 consecutive trials with no errors. Mary was seated in the dining room with all of the residents of the facility, at her own table in a wooden cubby where the black light was hung above her head to illuminate white food on the plate and a white cup on the table surface. The room was also accommodated for the trained staff to sit or stand so that they could facilitate the self-feeding activity. Accordingly, there were two important environmental supports that facilitated Mary’s new responsiveness: (a) the black light environmental condition and (b) procedures for self-feeding training. Black light conditions stimulated her visual-sensory awareness that triggered Mary’s attention and enhanced her self-awareness, which was measured by her participation in all task-related behaviors of self-feeding. Initially, white foods were added one at a time until there were four choices on a plate, each providing different textures, different tastes, and various nutritional components. 

Aides were trained in how to choose prompts from a hierarchy of physical, verbal, gestural, and demonstration assistance, which were faded on each trial on every step of the spoon to mouth and drinking from a cup. The fading and timing process of prompting was created to allow Mary to reach her highest level of independence at each step over time until she mastered every step and developed a new habit of self-feeding under black light conditions. The beauty of the TAW process was that after establishing the habit of self-feeding (which took 90 days to develop) and encouraging aides to delay the standby assistance to give Mary time to problem-solve and initiate her own process, standby assistance was faded as well until Mary developed the habit of coming to her private space alone, maintained the habitual response of eating until food and drink were gone, and push in her chair when everyone exited the dining room with no assistance.

## Discussion

The focus of this paper was to highlight the generalizability of the black lighting approach to heighten sensory awareness, a personal factor that enabled the child to interact with objects. However, the training seemed to sustain her interaction with related objects, whereby she acquired another self-help functional task. Using an innovative approach to make a lighting adjustment, this study suggests that the environment is as important as personal factors in establishing new habitual behavioral patterns of children with ASD. These interactive components were key to understanding how this child assigned meaning to eat under black light conditions. Black light prompted her attention to the objects brought to her attention in the foreground. Training under the black light condition maintained her attention and established the repertoire and habit to engage in a critical daily self-care life skill.

Under the impetus of black light conditions set up to facilitate self-feeding, Mary was attracted to and perceived self-feeding as a meaningful and pleasing task. Therefore, she expanded no choice to more complex choices. The child's response to the duplicable and consistent training elicited her brain meaning-making capacity, whereby she learned how to self-feed. It was her brain meaning-making process that helped her (a) to attract to the pleasing visual task, and the sensual experience of self-feeding, (b) adapt to learn the routine of interacting with objects in the environment, and (c) eventually master the task that perpetuated the learning and changed her behavioral tendency to include objects in her environment. On this basis, the child's tendency for the black light conditions was not a passive act but the culmination of intentional actions, whereby she directed her attention to a selected aspect of her environment and abstracted, interpreted, and learned, thus creating meaning and new perceptual state. These results corroborated the idea that a child’s tendency to approach a visual task could result from the interaction between the environmental light (which would be seen as a signal) and the meaning-making process in the brain (which may have impacted the child’s behavioral tendencies) (8). Overall, this research finding, while preliminary, suggests that the limited activity choice made by children with ASD could be altered by black lighting as an important aspect of the physical environment that accentuates objects in the foreground from a black background. Accordingly, to influence the child’s behavior and facilitate the meaning-making process in the brain, the use of backlighting served as a prelude to the creation of new activity choices. However, this needs experimental research on a larger sample to be empirically investigated and generalized to other children. 

## Author’s Contribution

EP involved in the conception and designing the study. Both authors performed the data analysis and interpretation. They also contributed to the drafting and revision of the article, as well as approval and submission of the final version**.**

## Conflict of Interest

The authors declare no potential conflicts of interest 
